# Digital Health Applications (DiGA) for Treating Depression and Generalized Anxiety Disorder: Protocol for a Systematic Health App Review and Systematic Review of Published Evidence

**DOI:** 10.2196/63380

**Published:** 2025-07-10

**Authors:** Annika Schmitz, Lucas Kueppers, Jacob Klein, Silke Frey, Arian Karimzadeh, Ana Luísa Neves, Birgitta Weltermann

**Affiliations:** 1 Institute of General Practice and Family Medicine University Hospital Bonn Bonn Germany; 2 Department of Primary Care and Public Health Imperial College London London United Kingdom

**Keywords:** digital therapeutics, mental illness, mental distress, psychological well-being, evidence synthesis, mental health, mHealth, digitalization, mobile health

## Abstract

**Background:**

Depression and generalized anxiety disorder (GAD) are widespread mental health diseases with significant individual and societal consequences. Psychotherapy, particularly cognitive behavioral therapy (CBT), is a common treatment approach, but its application is limited due to costs and staff shortages. Germany has been the first country to integrate and reimburse digital health applications (DiGAs) as an easily accessible treatment option since 2020. Despite regulatory processes, skepticism among physicians regarding clinical relevance and evidence persists.

**Objective:**

This protocol aims to describe the methodology of the planned systematic review. Using expert ratings, the app review will assess the guideline conformity, functions, and usability of German DiGAs for depression and GAD listed at the Federal Institute for Drugs and Medical Devices (BfArM). The additional systematic review will synthesize the effectiveness and quality of these DiGAs based on randomized controlled trials.

**Methods:**

The study protocol follows the 2015 PRISMA (Preferred Reporting Items for Systematic Reviews) guideline and was registered in the international Prospective Register of Systematic Reviews (PROSPERO). The review consists of 2 parts: (1) a systematic health app review of DiGAs addressing depression or GAD and (2) a systematic review of published evidence on these DiGAs. The systematic health app review comprises a summary of the DiGA features including the Institute for Healthcare Informatics (IMS) App Functionality Scoring System, a guideline conformity check, and the Mobile Application Rating Scale (MARS) assessment. The systematic review of published evidence is based on a systematic literature search in electronic databases (MEDLINE via PubMed, Cochrane Central Register of Controlled Trials [CENTRAL], Web of Science), as well as relevant websites. The approach includes an effectiveness evaluation, a risk of bias assessment using the Cochrane tool, Risk of Bias 2 (RoB2), and an overall quality evaluation using the Grading of Recommendations, Assessment, Development and Evaluation (GRADE) method.

**Results:**

The systematic literature search was conducted in July 2024 and August 2024, and an updated search is planned for November 2025. Data extraction, narrative synthesis, and evaluation of DiGA and corresponding studies are expected to be completed in spring 2026. The results will be presented using a PRISMA flow diagram and tables to display general information, risk of bias, and overall quality.

**Conclusions:**

The review will influence both the actual use and future developments of DiGAs. Good quality characteristics will enhance transparency and trust among physicians, while quality deficits provide options for improvement by manufacturers and governing institutions. Consequently, patients’ care with DiGAs may improve.

**Trial Registration:**

PROSPERO CRD42024557629; crd.york.ac.uk/PROSPERO/display_record.php?RecordID=557629

**International Registered Report Identifier (IRRID):**

PRR1-10.2196/63380

## Introduction

### Prevalence and Consequences of Depression and Generalized Anxiety Disorder

Depression and generalized anxiety disorder (GAD) are common disorders worldwide [1-5]. Approximately 15% to 20% of individuals experience depression at least once in their lifetime. Similar to other western nations, the 12-month prevalence of depression in Germany in 2015 was 9.7% for women and 6.3% for men, while the prevalence of GAD in Germany was 1.2% for men and 2.8% for women [6,7]. A significant proportion of patients have both diseases. Patients with GAD exhibited a 12-month prevalence rate of comorbid depression of 78.9% [8]. High prevalence rates of depression lead to significant individual and systemic consequences, including reduced quality of life, increased morbidity with a rise in chronic diseases, higher service utilization, and more sick leave days, as well as reduced work performance and early retirement [9-11].

The common therapeutic approach for depression and GAD is psychotherapy, especially cognitive behavioral therapy (CBT), which takes up a lot of manpower [12-14]. CBT “utilizes behavioral and cognitive strategies, particularly exposure, cognitive restructuring, changes in behavior, and development of coping skills, to address learned and conditioned behaviors, thoughts and emotional and psychophysiological reactions” [15]. Due to a shortage of professionals, long waiting times are prevalent in Germany. Digital health applications (DiGAs) were introduced in 2020 in the German health care system. DiGAs can bridge the waiting time for therapy or even replace human-guided face-to-face therapy by using CBT strategies [15,16].

Internet-based CBT (iCBT), as provided by DiGAs, can be divided into unguided and guided iCBT [17]. An international systematic review of applications using unguided and guided iCBT demonstrated their effectiveness [18]. “Unguided iCBT has considerable potential for improving long-term results of interventions with constrained economic and workforce resources” [18]. However, the intervention content differs between apps, and research is needed to identify the intervention components that are most effective [17,19].

### DiGAs in Germany

Germany was the first country to introduce an institutionally regulated approval procedure for mobile health apps, known as DiGAs [20]. DiGAs are “based on digital technologies” [21] and used by patients (with or without supervision by a health care provider) to support “the recognition, monitoring, treatment or alleviation of diseases or the recognition, treatment or alleviation or compensation of injuries or disabilities” [21] but not to prevent diseases. These DiGAs must provide scientific evidence of their effectiveness, distinguishing them from conventional apps available in app stores, which do not undergo regulatory review nor require proof of efficacy.

DiGAs are part of the digital transformation of the German health care system and regulated by legal provisions such as the Social Code – Book V – Statutory Health Insurance (SGB V) including the Digital Care Act and the Digital Act [21-23]. They are intended to enable evidence-based, low-threshold, and personalized health care and complement existing treatment services.

To be an official reimbursable DiGA, the device must be listed in the DiGA directory of the Federal Institute for Drugs and Medical Devices (BfArM) [21,24]. The BfArM holds regulatory authority and is responsible for the scientific evaluation of the efficacy and health care benefits of DiGAs. Manufacturers must apply to the institute for DiGA listing. A fundamental prerequisite for a DiGA listing is its classification as a registered medical device of risk class I, IIa, or IIb according to the Medical Device Regulation [23,25]. In order to be listed as an official DiGA, the BfArM has stipulated several requirements, including security, functionality, quality, data security, and data protection, that must be fulfilled according to Section 139e of the SGB V [21]. A key aspect of the approval process is the demonstration of medical benefits or patient-relevant structural and procedural improvements through clinical trials or other validated scientific methods. Economic indicators or reductions in health care personnel workload alone are not sufficient to justify approval [21].

To ensure rapid access to digital health care solutions, a fast-track procedure has been implemented, ensuring a listing decision within 3 months [26]. Depending on the available evidence, listing in the BfArM DiGA directory can be either permanent or provisional. Provisional admission to the directory is linked to a trial phase in which a positive therapeutic effect must be demonstrated within 12 months to obtain permanent approval [21]. After successful evaluation, DiGAs become reimbursable, both in permanent and temporary status. Physicians and psychotherapists can prescribe DiGAs nationally, at the expense of statutory health insurance or private insurance companies, similar to pharmacological agents. Patients can also apply to their health insurance company to use a DiGA. For this, patients need a qualifying diagnosis that entitles them to use it. Currently, 59 DiGAs are listed, including 19 provisionally and 40 permanently approved applications. Additionally, 10 DiGAs have been removed from the list due to insufficient evidence or voluntary withdrawal by manufacturers (as of March 2025) [24].

Despite the requirements, physicians criticize the process and question the effectiveness of DiGAs, especially in treating depression [12,27]. In a 2022 online survey with approximately 2600 physicians, 27.7% considered DiGAs for depression to be ineffective or contraindicated [27]. Additionally, a lack of information regarding DiGA content and effectiveness was noted. Strong evidence is considered a factor that facilitates physicians’ readiness to prescribe DiGAs; however, this is frequently lacking [27,28]. Critics particularly highlight the absence of study and analysis plans, which are required to be submitted to the BfArM but are not mandated for publication. In terms of methodology, the high dropout rates and lack of blinding in the studies are criticized. The criticism also extends to the selection of end points, with calls for a greater emphasis on patient-centered structural and procedural end points that align more closely with real-world evidence. This warrants the evaluation of DiGAs using patient-reported outcome measures and patient-reported experience measures [29-32].

### Rationale for Performing This Systematic Review

Only a few studies have systematically evaluated DiGAs, focusing on various aspects. Kolominsky-Rabas et al [31] analyzed the methodological quality of studies related to DiGAs for mental health and the nervous system and concluded that not all studies fulfill good scientific practice criteria. Lantzsch et al [29] examined clinical trials on DiGAs and reported a high risk of bias, especially regarding the reporting quality. A scoping review of DiGAs for treating mental illnesses by Schreiter et al [32] focused solely on summarizing clinical evidence but did not address care guidelines, usability items, or a risk-of-bias evaluation. Haaf et al [33] performed a systematic review and meta-analysis “to estimate the immediate effects and the long-term effects” of freely available applications and DiGAs for treating depression. They performed sensitivity and subgroup analyses but no content analysis nor usability evaluation [33]. The international Prospective Register of Systematic Reviews (PROSPERO) lists 2 systematic review protocols about DiGAs. Nevertheless, there is no overlap because the authors focused either on DiGAs for overweight and obesity or solely on the risk of bias of all DiGAs [34,35]. The review approach described in this protocol is designed to be more comprehensive by including all available evidence, current literature, a comparison with guideline-recommended content, and usability testing.

### Review Objective and Question

The anticipated systematic review aims to synthesize, through a structured methodological approach, the best available evidence concerning the quality of randomized controlled trials (RCTs) of DiGAs designed to support patients with depression or GAD. Specifically, the review seeks to examine the effectiveness of DiGAs on psychological well-being in comparison with control conditions, as well as to assess the risk of bias in the included RCTs. Additionally, the review aims to analyze the intervention strategies used by DiGAs in relation to national guidelines for the treatment of depression and GAD and to evaluate their usability and functional quality based on expert assessments.

## Methods

### Overview

**Figure 1:**
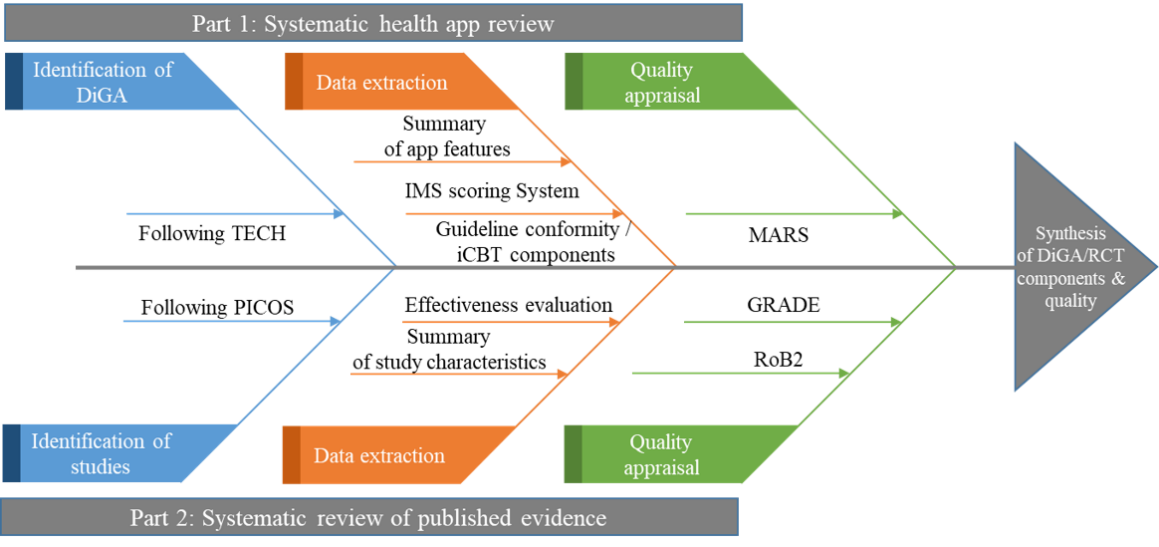
Review Process.

The systematic review is structured in 2 parts: (1) systematic health app review evaluating the selected DiGAs and (2) systematic literature review evaluating effectiveness studies of the DiGAs. Both parts evaluate the DiGAs identified in Part 1. The review process is illustrated in Figure 1. This review addresses guideline conformity, quality, and functions of the DiGAs as well as the published evidence regarding DiGAs’ effectiveness. The systematic review will be conducted and reported in accordance with the 2020 PRISMA (Preferred Reporting Items for Systematic Review and Meta-Analysis) checklist [36]. The systematic review protocol is registered in PROSPERO (CRD42024557629). If unforeseen changes are needed, we will document them by adding an amendment to the registry.

### Part 1: Systematic Health App Review

#### Identifying Apps

The planned systematic health app review will be conducted and reported in accordance with the methodological considerations of Gasteiger et al [37]. This involves a 2-stage approach: The first stage entails screening the app’s title and description on the app store, and the second stage involves downloading the app and assessing its eligibility.

Adaptations in the search strategy are required, because the review’s focus is on reimbursable DiGA not on freely available apps in the common app stores. Thus, DiGA will be identified via the DiGA directory not in app stores.

The BfArM DiGA directory is the primary and only source used to identify DiGAs, as it contains the complete list of DiGAs. The BfArM directory was filtered using the category “mental health” and the status “permanently listed” [24]. Listing status is important because only permanently listed DiGAs are obliged to have published evidence. The search was conducted in July 2024, and DiGAs identified stem from 2020 (earliest DiGA inclusion possible at the BfArM) through 2024. An updated search will be conducted shortly before the review is published (approximately in November 2025). After this initial selection, the 2-step approach was used.

Two reviewers performed and documented the screening process. The inclusion criteria were selected in accordance with the TECH (Target user groups, Evaluation focus, Connectedness, Health domain) framework (Textbox 1) [37]. The app search and screening process will be displayed as a flow diagram. Given that access to DiGAs is regulated via a doctor’s prescription or a health insurance application, the authors will contact the DiGA manufacturers to request access to DiGAs.

**Inclusion and exclusion criteria following the TECH (Target user groups, Evaluation focus, Connectedness, Health domain) framework [37].**

Target user groups

- The app must address adults aged 18 years and older.
- Target users must currently have depression or generalized anxiety disorder (GAD).
- There are no criteria for gender, age, nor ethnicity.
- Apps not specifically designed for adults (≥18 years) or those that do not target individuals currently with depression or GAD will be excluded.

Evaluation focus

- The evaluation centers on app characteristics, particularly the psychotherapeutic approach.
- The quality and usability of the app will be evaluated.
- The app needs to meet standards to support individuals with depression or GAD.
- Apps that do not use a psychotherapeutic approach or fail to meet the defined standards of quality and usability will not be considered.

Connectedness

- Standalone applications that operate independently from other technologies are considered.
- However, apps that provide the option to connect with other technologies are not excluded.
- Apps that cannot function independently are excluded.

Health domain

- Apps need to support patients with at least one of the following International Classification of Diseases (ICD) indications: F32.0; F32.1; F32.2; F33.0; F33.1; F33.2; F34.1; F41.1.
- Digital health applications (DiGAs) are excluded if they address mainly other mental disorders, such as agoraphobia, panic disorder, nonorganic vaginism and dyspareunia, nonorganic insomnia and sleep disturbance, and mental and behavioral disorders caused by alcohol, tobacco, or other drugs.

#### Data Extraction

The DiGAs will be evaluated using 4 different strategies including frameworks and guidelines: (1) a tabular summary of the features as suggested by Arnhold et al [38], (2) the Institute for Healthcare Informatics (IMS) App Functionality Scoring System [39], (3) a comparison of the DiGA content with professional guidelines for both diseases and disease-specific iCBT components published in the literature [12,14,40-43], and (4) a quality appraisal using the Mobile Application Rating Scale (MARS) [44].

#### Tabular Summary

Arnhold et al [38] developed a summarizing checklist for their study on mobile apps for weight loss. Modifications were implemented to align with DiGAs for mental well-being. The form includes details regarding general information, operating system, acquisition costs, and data storage. Range of functions is excluded because a functionality assessment for consumer health care apps is used. Two reviewers are responsible for extracting data from the included DiGAs using this form and resolving any disparities through discussions with a third reviewer.

#### Functionality Assessment

Based on the recommendations of Gasteiger et al [37], the IMS App Functionality Scoring System is used to assess the functionality of the DiGAs [39]. The assessment comprises 7 functionality criteria and 4 subcriteria (Table 1). The focus is on the availability of the functions, thus the reviewer rate the functionality using a binomial 0 for not available or 1 for available. A visual representation of the findings in the form of a radar graph is planned. Interrater reliability is calculated using the Cohen kappa statistic [37,39].

**Table 1:** Institute for Healthcare Informatics Functionality Scoring System [39].

Functionality scoring system^a^	Description
1. Inform	Provides information in a variety of formats (text, photo, video)
2. Instruct	Provides instructions to the user
3. Record	Captures user-entered data
3a. Collect data	Able to enter and store health data on individual phone
3b. Share data	Able to transmit health data
3c. Evaluate data	Able to evaluate the health data entered by patient and provider, provider and administrator, or patient and caregiver
3d. Intervene	Able to send alerts based on the data collected or propose behavioral intervention or changes
4. Display	Graphically displays user-entered data and output user-entered data
5. Guide	Provides guidance based on user-entered information, may further offer a diagnosis, or recommend a consultation with a physician or a course of treatment
6. Remind or alert	Provides reminders to the user
7. Communicate	Provides communication between health care professionals and patients or provides links to social networks

^a^Total score (0-11): 1 point is assigned to each functionality that is present.

#### Comparison With Guidelines

To evaluate the guideline conformity of the DiGAs, 2 checklists were prepared from the National Care Guidelines for Depression and the German S3 Guideline for Anxiety Disorders [12,14]. These checklists summarize the recommended therapy approaches. In addition, iCBT components for depression and GAD were extracted from the literature and compiled in a checklist [40-43]. iCBTs that were not applicable were excluded (eg, waiting component, conventional drug treatment, placebo effect). During the review, reviewers will mark all DiGA content modules as “yes” (1), “no” (0), or “to be discussed.” In addition, free text options are offered (see Multimedia Appendix 1). The compiled findings will be used to generate an overview of the DiGAs’ content.

To ensure a high level of objectivity, 6 patient cases based on the literature will be used for all raters involved, namely 3 cases of depression and 3 cases of GAD (see Multimedia Appendix 2) [40-44]. The patients presented differ in severity, age, and prognosis. All checklists were piloted by 2 physicians and a health care scientist using a web-based application for depression. The form was then discussed with a psychiatrist treating patients with depression and GAD.

#### Quality Appraisal

The 2 reviewers will evaluate the app quality using the MARS tool. MARS is a reliable and valid tool for appraising mobile health interventions, with 19 items evaluating 4 dimensions: engagement, functionality, aesthetics, and information. Interrater reliability will be analyzed using the intraclass correlation coefficient [37,44].

### Part 2: Systematic Review of Published Evidence

This protocol is reported in line with the 2015 PRISMA-P (Preferred Reporting Items for Systematic Review and Meta-Analysis Protocols) checklist, and the completed PRISMA-P checklist is provided in Multimedia Appendix 3 [45]. During the review process, 2 reviewers performed the literature search and will extract and evaluate the data. In case of disagreement, a more experienced reviewer will lead discussions until a consensus is reached. Cohen kappa is used to calculate the agreement coefficient [46].

#### Identifying Studies

After identifying the DiGAs, a systematic literature search was conducted in July 2024 and August 2024. An updated search is planned shortly before the review is published (approximately in November 2025). This process was divided into 3 steps. First, a systematic literature search was conducted in the electronic bibliographic databases MEDLINE via PubMed, the Cochrane Central Register of Controlled Trials (CENTRAL), and Web of Science. Only studies in German or English were considered. All citations identified by the search strategy were managed using Rayyan, which was used to remove duplicate records and further screen the results. There were no restrictions regarding the publication year. Although DiGAs were implemented in the German health care system in 2020, studies of these might have been carried out earlier.

A separate search using the original DiGA name was conducted for each identified DiGA. Search queries must be customized to suit the specific requirements of various databases and electronic libraries using Boolean logic and Medical Subject Headings (MeSH) terms. The search terms focused on 3 key areas: DiGA name, impact evaluation, and research design. To ensure a comprehensive literature search on the effectiveness of the selected DiGAs, we used a detailed and structured search string. For instance, the search string for one DiGA in PubMed is listed in Table 2.

**Table 2:** Search string for study identification.

Number	Field of interest	Search string
1	DiGA^a^ name	Deprexis
2	Impact evaluation: studies that focus on the effects or effectiveness of the intervention	(“effect”[All Fields] OR “effecting”[All Fields] OR “effective”[All Fields] OR “effectively”[All Fields] OR “effectiveness”[All Fields] OR “effectivenesses”[All Fields] OR “effectives”[All Fields] OR “effectivities”[All Fields] OR “effectivity”[All Fields] OR “effects”[All Fields] OR “effectiv*”[All Fields])
3	Impact evaluation: Studies that focus on the safety of the intervention	(“patient safety”[MeSH Terms] OR (“patient”[All Fields] AND “safety”[All Fields]) OR “patient safety”[All Fields] OR (“patient harm”[MeSH Terms] OR (“patient”[All Fields] AND “harm”[All Fields]) OR “patient harm”[All Fields]) OR “safety management”[MeSH Terms])
4	Impact evaluation: Studies that focus on equity regarding the intervention	(“equities”[All Fields] OR “equity”[All Fields] OR “disparit*”[All Fields] OR “inequit*”[All Fields] OR “inequalit*”[All Fields])
5	Research design	(“studies”[All Fields] OR “study”[All Fields] OR “study s”[All Fields] OR “studying”[All Fields] OR “studys”[All Fields] OR (“research personnel”[MeSH Terms] OR (“research”[All Fields] AND “personnel”[All Fields]) OR “research personnel”[All Fields] OR “researcher”[All Fields] OR “researchers”[All Fields] OR “research”[MeSH Terms] OR “research”[All Fields] OR “research s”[All Fields] OR “researchable”[All Fields] OR “researche”[All Fields] OR “researched”[All Fields] OR “researcher s”[All Fields] OR “researches”[All Fields] OR “researching”[All Fields] OR “researchs”[All Fields]) OR (“clinical trial”[Publication Type] OR “clinical trials as topic”[MeSH Terms] OR “clinical trial”[All Fields]) OR (“randomized controlled trial”[Publication Type] OR “randomized controlled trials as topic”[MeSH Terms] OR “randomized controlled trial”[All Fields] OR “randomised controlled trial”[All Fields]))
6	Combined string	#1 AND #5 AND (#2 OR #3 OR #4)

^a^DiGA: digital health applications.

Second, a gray literature search was conducted in the BfArM directory, corresponding websites, and manufacturers’ websites. Third, the snowball method was used to identify further studies that evaluate the DiGAs of interest. This includes a search for studies of the DiGAs published under a different name.

All identified titles and abstracts were independently screened by 2 reviewers. Disagreements were documented and discussed between the reviewers until a joint decision was made. In the next step, the full texts of the eligible studies will be screened and rated following the processes described. The complete process will be displayed in a PRISMA flow diagram.

#### The Eligibility Criteria

The criteria shown in Textbox 2 were used to identify eligible studies. The eligibility criteria are based on the population, intervention, comparison, outcomes, and study design (PICOS) scheme [47].

**Eligibility criteria in the population, intervention, comparison, outcomes, and study design (PICOS) scheme.**

Population

- The examined participants must not meet any criteria in terms of gender, age, or ethnicity.
- Symptoms of depression or generalized anxiety disorder (GAD) must be present for the participants.
- To identify a broad range of studies examining digital health applications (DiGAs), all countries are included.
- Excluded are studies focusing on participants who do not present psychological symptoms.

Intervention

- The study must examine at least one of the predefined DiGAs.

Comparison

- The comparison group must receive either the standard therapy, no therapy, or therapy with another DiGA, based on the specifications of the Federal Institute for Drugs and Medical Devices (BfArM).
- The comparison group must be as close as possible to treatment as usual.

Outcomes

- Included types of outcome measurements are any objective measurements of health that are directly linked to the patient (eg, depressive symptom severity, activities of daily life, cognitive outcomes relating knowledge, and others).
- Excluded are outcomes that focus solely on the physician or the economy.

Types of studies

- Studies must be conducted as a randomized controlled trial to ensure a rigorous assessment of DiGA effectiveness under controlled conditions.

#### Data Extraction From RCTs

Data extraction will be performed using prepiloted forms. Missing data will be requested from the study authors or DiGA manufacturers. Two reviewers will independently extract the data. Similar to the screening process, an experienced reviewer will discuss any disagreements.

All included studies will be summarized in a table. Table items include DiGA name, author, year, title, inclusion criteria, exclusion criteria, indication, recruitment, treatment, gender and age distribution of the study population, intervention and control, outcomes and operationalization, study design, randomization, blinding, sample size calculation and sample size, dropouts, results, observation period, observation times, place of study, time of study, strengths, and limitations.

#### Effectiveness

We will evaluate the effectiveness of the DiGAs by calculating risk ratios for dichotomous data or standard mean differences for continuous data, utilizing data extracted from published studies or data obtained directly from study authors. Heterogeneity among the studies will be assessed using the I^2^ test, which is known for its robustness to sample size variations. To ensure the robustness of our findings, sensitivity analyses will be conducted to examine the impact of different assumptions or methodological choices on our results.

Furthermore, we will address potential publication bias by generating funnel plots and conducting additional analyses using the Egger test. This multifaceted approach will provide a comprehensive evaluation of the intervention’s effectiveness while accounting for potential sources of bias and variability across the included studies [47].

#### Quality Appraisal

Risk of bias will be assessed independently by 2 reviewers using the Cochrane tool to assess the risk of bias in randomized trials (Risk of Bias 2 [RoB2]) [48]. The RCTs are rated in the following categories: randomization sequence generation, treatment allocation concealment, blinding, completeness of outcome data, selective outcome reporting, and other sources of bias. Again, a third reviewer will lead any discussions about disagreements. The level of the risk of bias will be presented in the final review.

The overall quality of evidence will be assed using the GRADE (Grading of Recommendations, Assessment, Development and Evaluation) method [49]. This method helps rate the quality of the evidence. The rating process will be conducted in the same manner as the risk of bias assessment.

## Results

### Status

The systematic literature search was conducted in July 2024 and August 2024. The process involved searching the electronic bibliographic databases MEDLINE via PubMed, CENTRAL, and Web of Science. Only studies published in German or English were considered. All citations identified with the search strategy were managed using Rayyan, where duplicate records were removed and initial screening was performed. An updated search is planned in November 2025 to capture any new evidence before the review is submitted.

The subsequent steps, including data extraction, narrative synthesis, evaluation of DiGAs, and corresponding studies, as well as the final writing of the systematic review and meta-analysis are expected to be completed in spring 2026.

### Results of Systematic Health App Review

In a scoping process, 26 DiGAs for mental health were identified, 17 of which are permanently listed [24]. The permanent inclusion in the DiGA directory of the BfArM requires publication of the positive therapeutic effect of a DiGA.

For the DiGA evaluation, tables will be generated to present general information and intervention components, along with quality ratings for each DiGA. Interrater reliability calculations will be presented. These results will provide a comprehensive comparison of DiGAs for depression and GAD focusing on content and quality.

### Results of Systematic Review of Published Evidence

A PRISMA flow diagram will be used to illustrate the inclusion process. The results of the study evaluation will be presented in tables to show general information, risk of bias, and overall quality. Interrater reliability calculations will be presented.

### Integrated Data Synthesis Across Both Review Components

Both components of the review are anticipated to yield valuable insights into DiGAs. To complement this, a combined synthesis through subgroup analyses is planned to assess how specific qualitative and content-related features relate to intervention effectiveness within the meta-analytic findings.

## Discussion

### Overview

In contrast to systematic literature reviews, there is no gold standard for conducting or reporting systematic health app reviews yet. As more apps are brought to market and depression rates rise, there is a need for such a standard to ensure high-quality care. Despite the evident benefits of DiGAs, several issues persist. Methodological flaws in efficacy studies, including inadequate participant screening, reliance on self-reported outcomes, and the use of generalized content approaches undermine their reliability [12,29,32,33,50]. Addressing these concerns through rigorous content, quality, and evidence-based investigations is crucial for advancing the evidence of digital health interventions.

Since the integration of DiGAs into the German health care system in 2020, patients have been able to access these digital tools easily, receiving support for a variety of medical conditions. The majority of DiGA activations have been in the field of psychological and behavioral disorders. The use of digital mental health interventions has the potential to alleviate strain on the health care system, particularly in light of the growing shortage of skilled therapists [51]. However, concerns regarding the high costs associated with DiGAs persist [52].

During the trial phase, manufacturers independently determine the price of a DiGA, which is subsequently reimbursed by health insurance providers. Once a DiGA receives permanent listing status, its price is negotiated and set by an arbitration board. For instance, the cost of Velibra, a DiGA designed for anxiety disorders, was reduced by 51.70% following the trial phase—from €476 to €230 [52]. As price reductions are effective after the trial period, health insurance funds face substantial financial burdens in the initial phase. Conversely, manufacturers experience financial constraints when prices are reduced after permanent listing.

DiGAs have become well integrated into the German health care system, which is currently undergoing a digital transformation. Plans are in place to improve interoperability with other digital systems. The electronic patient record is currently being implemented, and interfaces with DiGAs are expected to be established. As a result, DiGAs must already comply with the necessary technical requirements. Despite these technical advancements, real-world implementation remains challenging. Physicians, for instance, face barriers to prescribing DiGAs, such as limited information and a lack of trust in their effectiveness. A current discussion revolves around the introduction of an application-accompanying success measurement [53]. Such an approach could, on the one hand, allow reimbursement to be based on measurable outcomes, thereby potentially reducing costs. On the other hand, it could provide physicians with better insights into the effectiveness of DiGAs for their patients, ensuring greater transparency in digital health care.

In general, it is likely that individuals with greater technological proficiency experience fewer difficulties with using these applications than those who are less tech-savvy. However, health literacy and patients’ self-management skills are crucial factors in successful DiGA adoption [54]. Therefore, in addition to the systematic evaluation of the evidence, further studies are needed to investigate DiGAs and, above all, the users.

### Limitations

The detailed analytic approach comprising an app review in addition to a systematic review addressing the content, functionality, and quality of DiGAs is one of the strengths of this review. However, it includes apps available only in Germany, which may limit the generalizability of the findings. The available evidence may restrict the effectiveness of the analyses in the review.

### Conclusions

Given the increasing availability and use of DiGAs for mental health, the anticipated multifaceted review will provide important insights into their current potential and quality for depression and GAD: The more is known about the quality of a DiGA, the better its actual benefit can be valued by physicians. High quality leads to greater trust and better utilization. Accordingly, indications of low quality would lead to specific recommendations for action. Ultimately, the quality of patient care is improved as DiGAs either gain more trust or targeted recommendations for improvement are made.

The review’s results will be considered in various contexts to generate actionable improvement suggestions. If additional evidence gaps are identified, proposals for further research will be made. The DiGA development process will be evaluated, particularly regarding content, with clinical perspectives included to validate or challenge physicians’ assumptions about DiGA effectiveness. The political dimension will assess the spread of DiGAs and potential future challenges. Finally, the results will be compared with international and national findings in this research area to contextualize insights and contribute to the development of therapies and access options for patients with depression and GAD.

## Appendix

Multimedia Appendix 1 Checklists for DiGA treating depression or GAD.

Multimedia Appendix 2 Cases for DiGA evaluation.

Multimedia Appendix 3 PRISMA-P checklist.

## Abbreviations

BfArM: Federal Institute for Drugs and Medical Devices (Bundesinstitut für Arzneimittel und Medizinprodukte)
CBT: cognitive behavioral therapy
CENTRAL: Cochrane Central Register of Controlled Trials
DiGA: Digital health applications (Digitale Gesundheitsanwendung)
GAD: generalized anxiety disorder
GRADE: Grading of Recommendations, Assessment, Development and Evaluation
iCBT: internet-based cognitive behavioral therapy
IMS: Institute for Healthcare Informatics
MARS: Mobile Application Rating Scale
MeSH: Medical Subject Headings
PICOS: population, intervention, comparison, outcomes, and study design
PRISMA: Preferred Reporting Items for Systematic Reviews
PRISMA-P: Preferred Reporting Items for Systematic Review and Meta-Analysis Protocols
PROSPERO: Prospective Register of Systematic Reviews
RCT: randomized controlled trial
RoB2: Risk of bias tool 2
SGB V: Social Code – Book V (Fünftes Buch Sozialgesetzbuch)
TECH: Target user groups, Evaluation focus, Connectedness, Health domain
