# Supplementary material for: Digital Health Applications (DiGA) for Treating Depression and Generalized Anxiety Disorder: Protocol for a Systematic Health App Review and Systematic Review of Published Evidence
Source: JMIR Res Protoc. 2025 Jul 10;14:e63380. doi: 10.2196/63380 (PMC12290428; doi:10.2196/63380)
Supplement: Multimedia Appendix 1 [file resprot_v14i1e63380_app1.pdf]

This is a Multimedia Appendix to a full manuscript published in JMIR Research Protocols.

1 Evaluation Sheet Depression

1.1 General Information

(adapted from Arnhold et al. (2014) [1])

Category	Subcategory	Text
**General information**		
	App name	
	App language(s)	
	Date of release (DiGA directory)	
	Risk classification (MDR)	
	Date of last update	
	Availability of a desktop application	
	Availability of a mobile phone application	
	Used Version number	
**Operating system**		
	App exclusively for the iOS operating system	
	App exclusively for the Android operating system	
	App for both operating systems available	
**Developer information**		
	Name of the developer	

This is a Multimedia Appendix to a full manuscript published in JMIR Research Protocols.

**Acquisition costs**		
	Full version	
**Target user groups**		
	Patients	
	Patients and physicians	
	Indications	
	Contraindications	
	Female	
	Male	
	Non-binary	
	Age	
**Interfaces**		
	Availability of an interface/connectivity to an external sensor(s)/device	
**Data storage**		
	Whom belongs the data?	
	Where is data stored?	
	How long is it stored? (general data affiliations)	
**Security**		
	Password	
	Two-factor authentication	
	Additional security	

This is a Multimedia Appendix to a full manuscript published in JMIR Research Protocols.

1.2 Intervention Components

(extracted from NVL [2], Watkins et al. (2023) [3] , Furukawa et al. (2018/2021) [4,5])

Component	Category	Item	Subcategory	Yes	No	tbd*
1. Psychoeducation and training	1.1 Information about the disease	1.1.1	Symptoms			
		1.1.2	Diagnosis			
		1.1.3	Cause(s)			
		1.1.4	Course			
		1.1.5	Biological, psychological and psychosocial aspects			
		1.1.6	Treatability and prognosis			
		1.1.7	Communication of a bio-psycho-social disease model to relieve patients of feelings of guilt, failure and self-blame			
	1.2 Information on self-management	1.2.1	Connection between thinking, behaviour and feelings			
		1.2.2	Sleep hygiene			
		1.2.3	Anxiety management			
		1.2.4	Stress management			
		1.2.5	Communication and conflict resolution skills			
		1.2.6	Day and lifestyle structuring (e.g. developing enjoyable activities)			
		1.2.7	Learning relaxation techniques			
		1.2.8	Dealing with the illness in a work context			
	1.3 Information about the treatment	1.3.1	Mode of action, expected effects, possible interactions and side effects of antidepressants, latency, need for maintenance therapy, discontinuation symptoms and rebound phenomena			
		1.3.2	Psychotherapy methods, effectiveness, need for continuation after symptoms have subsided, possible positive and negative side effects of psychotherapy			
		1.3.3	Supporting services (sports and exercise therapies, light therapy, sleep deprivation therapy, etc.)			
		1.3.4	Structured/complex forms of care			
		1.3.5	Options in the event of non-response to treatment			
		1.3.6	Neurostimulatory procedures			
		1.3.7	Psychosocial therapies			
		1.3.8	Benefits and potential harm of self-help groups			
		1.3.9	Benefits of involving relatives and their involvement in relatives' groups			
		1.4.1	Medical rehabilitation			

	1.4 Information on rehabilitative services, benefits for participation and other services	1.4.2	Benefits for participation in work and education, benefits for social participation			
		1.4.3	Services to support children, young people and families (household help, educational assistance, etc.)			
		1.4.4	Information on counselling options regarding these services			
	1.5 Relapse prevention and crisis management	1.5.1	Recognizing relapses and crises			
		1.5.2	Dealing with suicidal tendencies			
2. Cognitive behavioural therapy (CBT)		2.0.1	Individual problem analysis and therapeutic interventions that correct the problem behavior of depressed patients and develop an improved problem-solving repertoire			
		2.0.2	Cognitive approaches aimed at alleviating depressive symptoms by identifying and changing dysfunctional attitudes and thinking and behavioral schema goals			
	2.1 Acceptance and Commitment Therapy (ACT)	2.1.1	Behavioural therapy techniques combined with mindfulness- and acceptance-based strategies and interventions to clarify values			
	2.2 Mindfulness Based Cognitive Therapy (MBCT)	2.2.1	Cognitive behavioural therapy techniques with mindfulness exercises			
	2.3 Cognitive Behavioural Analysis System for Psychotherapy (CBASP)	2.3.1	Combines cognitive, behavioural, interpersonal and psychodynamic strategies			
3. Psychonalytically based procedures[4]		3.0.1	Patients reflect on their feelings within a regression-promoting setting, primarily through "free speech"			
		3.0.2	During treatment, psychotherapists observe their own, patient-related inner experience to better understand the patient and empathize with their inner experience, feelings and fears, verbalize them and address feelings that are unacceptable to the patient.			
		3.0.3	Depressive experiences as well as concrete conflict actualizations in the current life situation			
		3.0.4	Inner conflicts should be made conscious and the patients' abilities to overcome them should be used and strengthened.			

4. Systemic therapies[3]		4.0.1	Focus on the social context of mental disorders			
		4.0.2	Members of the social system that is important to the patient are often directly or indirectly involved in the treatment			
		4.0.3	Using the strengths and resources of patients and relatives to change unfavourable behaviours, interaction patterns and evaluations, to support helpful communication and relationship patterns and to jointly develop solutions to existing problems and conflicts			
		4.0.4	Circular or solution- and resource-oriented questions, paradoxical interventions or methods that explore the relationship experience of patients and their relatives			
5. Cross-procedural impact factors for depressive disorders		5.0.1	Establishment of a sustainable therapeutic "working alliance"			
		5.0.2	Clarification of motivation, goals, methods and setting of the treatment			
		5.0.3	Determination of the individual treatment framework (implementation agreements, commitment of practitioners, duration, frequency and financing), taking into account the partial independence of this framework from ICD diagnoses			
		5.0.4	Recognizing suffering as a prerequisite for change			
		5.0.5	Experiencing unfavourable behavioural patterns within the therapeutic relationship and facilitating corrective learning experiences			
		5.0.6	Enabling a deeper emotional experience			
		5.0.7	Promotion of self-regulation and insight into relationship mechanisms and internal conflict and coping structures (especially avoidance of action and emotions)			
		5.0.8	Clarification of the partial functionality of the symptoms			
		5.0.9	Recognition and activation of personal and social resources (e.g. possible helpful actors in the patient's environment)			
		5.0.10	Initiating problem-solving and experiencing competence to promote self-esteem, self-congruence and self-efficacy, integrating what has been "understood" and "learned" into everyday life			
6. Monitoring		6.0.1	When using internet and mobile-based interventions, adherence and effectiveness should be regularly monitored.			
7. Therapeutic support		7.0.1	The use of Internet and mobile-based interventions should be accompanied by therapeutic support.			
8. Low-intensity interventions (NVL 4.2)	8.1 Bibliotherapy	8.1.1	Handing out self-help or self-management literature			
	8.2 Conversation-based interventions using psychotherapeutic techniques, e.g:	8.2.1	Psychoeducational-supportive conversations: explaining the illness; teaching simple self-help practices, e.g. regarding sleep-wake rhythm, daily structuring and anxiety management			
		8.2.2	Counselling: psychosocial discussion and intervention techniques			

		8.2.3	Problem-solving approaches: Teaching strategies for solving problems			
		8.2.4	Behavioural activation: Reduction of avoidance and protective behaviour, inactivity, anhedonia and social withdrawal			

*tbd = to be discussed

**iCBT[3–5]**	**No.**	**Yes**	**No**	**tbd***
Psychoeducation about depression	iCBT.1			
Cognitive restructuring	iCBT.2			
Behavioural activation	iCBT.3			
Interpersonal skill training	iCBT.4			
Problem solving	iCBT.5			

* tbd = to be discussed

1.3 Narrative Synthesis

Add additional therapy modules

This is a Multimedia Appendix to a full manuscript published in JMIR Research Protocols.

2. Evaluation Sheet DiGA – GAD

2.1 General Information

(adapted from Arnhold et al.(2014) [1])

Category	Subcategory	Text
**General information**		
	App name	
	App language(s)	
	Date of release (DiGA directory)	
	Risk classification (MDR)	
	Date of last update	
	Availability of a desktop application	
	Availability of a mobile phone application	
	Used Version number	
**Operating system**		
	App exclusively for the iOS operating system	
	App exclusively for the Android operating system	
	App for both operating systems available	
**Developer information**		
	Name of the developer	
**Acquisition costs**		
	Full version	

This is a Multimedia Appendix to a full manuscript published in JMIR Research Protocols.

**Target user groups**		
	Patients	
	Patients and physicians	
	Indications	
	Contraindications	
	Female	
	Male	
	Non-binary	
	Age	
**Interfaces**		
	Availability of an interface/connectivity to an external sensor(s)/device	
**Data storage**		
	Whom belongs the data?	
	Where is data stored?	
	How long is it stored? (general data affiliations)	
**Security**		
	Password	
	Two-factor authentication	
	Additional security	

This is a Multimedia Appendix to a full manuscript published in JMIR Research Protocols.

2.1 Intervention Components

(extracted from Checklist S3 Treatment of GAD_2021-06 [6], iCBT components (Titov et al.) [7])

Component	Category	Item	Subcategory	Yes	No	tbd*
1. Basic psychotherapeutic treatment		1.0.1	Active, flexible and, if necessary, supportive approach, conveying encouragement and hope			
		1.0.2	Empathic contact, building a trusting relationship			
		1.0.3	Exploration of the subjective disease model, clarification of the patient's current motivation and therapy expectations			
		1.0.4	Providing an understanding of the symptoms, their treatability and their prognosis, providing a "psychosocial disease model" to relieve the patient of feelings of guilt, self-blame and feelings of failure			
		1.0.5	Encouragement to deal with triggers of anxiety instead of avoidance			
		1.0.6	Clarification of current external problem situations, relief from currently overwhelming duties and demands at work and in the family situation			
		1.0.7	Support in formulating and achieving specific, achievable goals			
		1.0.8	Providing insight into the individual need for adequate therapies (e.g. medication, psychotherapy)			
		1.0.9	Involvement of relatives, strengthening resources			
2. Cognitive behavioural therapy (CBT)	1. Patients should learn:	2.1.1	Distinguishing between thoughts and emotions			
		2.1.2	To be aware that thoughts influence the occurrence of emotions and inappropriate behavior in sometimes unfavorable ways			
		2.1.3	That thoughts sometimes occur automatically without those affected realizing that their emotions are being influenced by them. In cognitive therapy, patients should learn to critically evaluate whether the automatically occurring thoughts are correct and/or helpful.			
		2.1.4	Develop skills to independently recognize, interrupt and correct dysfunctional (one-sided, erroneous) thoughts, but especially the underlying assumptions, in order to be able to behave more appropriately and adapt to the situation.			
	2. Psychoeducation	2.2.1	Providing information about the disorder, including frequently occurring worries and physical expressions of anxiety			
		2.2.2	Recommendation of suitable self-help materials			
	3. Cognitive and metacognitive approaches	2.3.1	Processing positive and negative metacognitions/meta-worries ("I worry that my worries will affect my health", "I can't control my worries", "If I worry, I'm better prepared")			

This is a Multimedia Appendix to a full manuscript published in JMIR Research Protocols.

		2.3.2	Reassess unrealistic assumptions about the benefits and drawbacks of worry			
		2.3.3	Develop a realistic assessment of the likelihood that problems will lead to negative consequences and how much suffering this will cause			
		2.3.4	Dealing with problems caused by intolerance towards feelings of insecurity and perfectionism			
	4. Exposure	2.4.1	In-sensu exposure to feared personal disasters and associated worries			
		2.4.2	Elimination of inappropriate safety behavior			
		2.4.3	Learning to tolerate fearful experiences instead of avoiding them			
	5. Reduction of safety behavior	2.5.1	Patients are encouraged to refrain from safety behaviors (e.g. reassurance calls from a mother asking if her children are healthy)			
	6. Emotion regulation	2.6.1	Relaxation methods			
		2.6.2	Strategies of acceptance and mindfulness			
	7. Problem solving techniques	2.7.1	Practicing problem-solving strategies to reduce inadequate solutions ("worrying")			
		2.7.2	Identification and reduction of avoidance behavior			
		2.7.3	Acquisition of interpersonal skills			
		2.7.4	Developing goals and life planning, carrying out enjoyable activities, increasing the perception of mental well-being			
	8. Relapse prevention	2.8.1	Preparation for periods in which new fears or events arise that are related to the prevailing worries			
3. Relaxation techniques		3.0.1	Progressive muscle relaxation			
		3.0.2	Applied Relaxation			
4. Analytical psychotherapy		4.0.1	Processing current conflict issues, centring on current symptoms, modifying neurotic object and self-representations, conscious thought patterns such as dysfunctional persuasion, problem-solving strategies, feelings, motives and affect regulation			
5. Depth psychology-based psychotherapy		5.0.1	Focused processing of current interpersonal conflicts and their symptom treatment			
6. Focal therapy		6.0.1	Treatment is strongly limited to the processing of one focus			
		6.0.2	Supportive-expressive psychotherapy (as analytical focal therapy)			
7.Eye Movement Desensitization and Reprocessing Therapy (EMDR)		7.0.1				
8. Sports		8.0.1	Therapeutic endurance training			

This is a Multimedia Appendix to a full manuscript published in JMIR Research Protocols.

9. Art therapies		9.0.1	music, dance, art, theatre and others			

* tbd = to be discussed

iCBT[7]	No.	Yes	No	tbd*
Relapse prevention	iCBT.1			
Worry exposure/behavioural experiments	iCBT.2			
Scheduled worry time	iCBT.3			
Educational material	iCBT.4			
Cognitive restructuring for thoughts, beliefs and meta-cognitive beliefs	iCBT.5			
Behavioural activation/activity scheduling	iCBT.6			
Interpersonal problem solving and/or communication skills training	iCBT.7			
Problem solving	iCBT.8			
Applied relaxation or other relaxation/de-arousal intervention	iCBT.9			
Mindfulness	iCBT.10			
Sleep management	iCBT.11			

*tbd = to be discussed

This is a Multimedia Appendix to a full manuscript published in JMIR Research Protocols.

2.3 Narrative Synthesis

Add additional therapy modules

This is a Multimedia Appendix to a full manuscript published in JMIR Research Protocols.

References

1. Arnhold M, Quade M, Kirch W. Mobile applications for diabetics: a systematic review and expert-based usability evaluation considering the special requirements of diabetes patients age 50 years or older. J Med Internet Res. 2014; 16:e104. Epub 2014/04/09. doi: 10.2196/jmir.2968 PMID: 24718852.
2. Bundesärztekammer (BÄK), Kassenärztliche Bundesvereinigung (KBV), Arbeitsgemeinschaft der Wissenschaftlichen Medizinischen Fachgesellschaften (AWMF). Nationale VersorgungsLeitlinie Unipolare Depression – Langfassung. Version 3.2.2022.
3. Watkins E, Newbold A, Tester-Jones M, Collins LM, Mostazir M. Investigation of Active Ingredients Within Internet-Delivered Cognitive Behavioral Therapy for Depression: A Randomized Optimization Trial. JAMA Psychiatry. 2023; 80:942–51. doi: 10.1001/jamapsychiatry.2023.1937 PMID: 37378962.
4. Furukawa TA, Efthimiou O, Weitz ES, Cipriani A, Keller MB, Kocsis JH, et al. Cognitive-Behavioral Analysis System of Psychotherapy, Drug, or Their Combination for Persistent Depressive Disorder: Personalizing the Treatment Choice Using Individual Participant Data Network Metaregression. Psychother Psychosom. 2018; 87:140–53. Epub 2018/05/30. doi: 10.1159/000489227 PMID: 29847831.
5. Furukawa TA, Suganuma A, Ostinelli EG, Andersson G, Beevers CG, Shumake J, et al. Dismantling, optimising, and personalising internet cognitive behavioural therapy for depression: a systematic review and component network meta-analysis using individual participant data. Lancet Psychiatry. 2021; 8:500–11. Epub 2021/05/03. doi: 10.1016/S2215-0366(21)00077-8 PMID: 33957075.
6. Bandelow B, Aden I, Alpers GW, Benecke A, Benecke C, Beutel ME, et al. Deutsche S3-Leitlinie Behandlung von Angststörungen. 2nd ed. ; 2021.
7. Titov N, Andersson G, Paxling B. ICBT in Psychiatry: Generalised Anxiety Disorder. In: Lindefors N, Andersson G, editors. Guided Internet-Based Treatments in Psychiatry. 1st ed. Cham, s.l.: Springer International Publishing; 2016. pp. 79–100.
