# Supplementary material for: Digital Health Applications (DiGA) for Treating Depression and Generalized Anxiety Disorder: Protocol for a Systematic Health App Review and Systematic Review of Published Evidence
Source: JMIR Res Protoc. 2025 Jul 10;14:e63380. doi: 10.2196/63380 (PMC12290428; doi:10.2196/63380)
Supplement: Multimedia Appendix 2 [file resprot_v14i1e63380_app2.pdf]

This is a Multimedia Appendix to a full manuscript published in JMIR Research Protocols.

1. Depression

Case Description – Depression

Case	Severity	Age	Process
A	severe	31-60 years-old	intermittent
B	mild	18-30 years-old	static
C	moderate	60–80 years-old	gradually improving

A) Mark – severe depression – intermittent process

(adapted from case Mark [1])

- 43-years-old male, divorced, living alone, joint custody of his twin adolescent daughters
- long history of depression, history of alcohol abuse
- recent episode of depression lasted without remission for 3 years
- had been in therapy previously
- periods of depression for “as long as I can remember”
- first episode of depression at age 12, shortly after his father abruptly left and severed all contact with Mark and his family (blamed himself for his father’s departure)
- never really happy
- depressed mood, loss of pleasure in nearly all activities, excessive guilt, fatigue, difficulty concentrating, and occasional passive thoughts of death
- he is spending most of his time alone, with the exception of caring for his daughters

B) Nancy – mild depression – static process

(adapted from case Nancy [2])

- 25-years-old, single
- editorial assistant to a well-known publisher
- graduated near the top of her class
- excellent social skills except at times she seemed overly compliant and timid
- generally cheerful, but mood shifts
- When talking about upsetting topics, particularly her relationship difficulties, she looked distressed and was close to tears
- depressive symptoms: sad, discouraged about the future, guilty, self-critical, crying, difficulty making decisions, difficulty getting anything done

C) Bernadette – moderate depression – gradually improving

(adapted from [3])

- married
- 70-year-old
- first episode of moderate depression
- retired from her position as a health care provider
- difficulties in time management, learning to manage money, and setting boundaries on her availability for baby-sitting her grandchildren
- never grieved properly for her sister's death

2. General Anxiety Disorder

Case description – GAD

Case	Severity	Age	Process
A	severe	31-60 years-old	intermittent
B	mild	18-30 years-old	static
C	moderate	60–80 years-old	gradually improving

A) Mark – severe general anxiety disorder – intermittent process

(adapted from case Mary [4])

- a 43-year-old man
- divorced with two children
- employed part time and cares for his mother who has Alzheimer's disease
- feeling 'stressed' all the time and constantly worries about 'anything and everything'
- tension in his shoulders, stomach and legs, his heart races and often he finds it difficult to breathe

B) Nancy – mild general anxiety disorder– static process

(adapted from case Nancy [2])

- 25-years-old
- editorial assistant
- Anxiety symptoms: anxiety, tension, fears of criticism and disapproval, palpitations, restlessness, tight muscles, rubbery legs, dizziness, headaches, fatigue

C) Phil – moderate general anxiety disorder - gradually improving

(adapted from [5])

- 70 years old
- biggest problem: worrying
- worries about "everything under the sun."
- equal worry about his wife who is undergoing treatment for breast cancer and whether he returned his book to the library -> bothered that both cause him similar levels of worry
- difficulty failing asleep, impatience with others, and significant back and muscle tension

References

1. Andrews G. Clinical Handbook of Psychological Disorders: a Step-By-Step Treatment Manual (4th edn) Edited by David H. Barlow. Guilford Press. 2007. US$75.00 (hb). 689pp. ISBN: 9781593855727. The British Journal of Psychiatry. 2009; 194:473. doi: 10.1192/bjp.bp.108.054650.
2. Persons JB. Essential components of cognitive-behavior therapy for depression. 1st ed. Washington, DC: American Psychological Association; 2001.
3. Miller MD, Wolfson L, Frank E, Cornes C, Silberman R, Ehrenpreis L, et al. Using interpersonal psychotherapy (IPT) in a combined psychotherapy/medication research protocol with depressed elders. A descriptive report with case vignettes. The Journal of Psychotherapy Practice and Research. 1997; 7:47–55. Available from: https://www.ncbi.nlm.nih.gov/pmc/articles/PMC3330480/.
4. National Institut for Health and Clinical Excellence (NHS). Generalised anxiety disorder and panic disorder (with or without agoraphobia) in adults. Clinical case scenarios for generalised anxiety disorder for use in primary care. NICE clinical guideline 113. ; 2011.
5. Society of Clinical Psychology Division 12 American Psychological Association. CASE STUDY Phil (Generalized Anxiety Disorder) [cited 15 Aug 2023]. Available from: https://div12.org/case_study/phil-generalized-anxiety-disorder/.
